# The Emerging Role of Tissue-Resident Memory CD8^+^ T Lymphocytes in Human Digestive Tract Cancers

**DOI:** 10.3389/fonc.2021.819505

**Published:** 2022-01-14

**Authors:** Xinyu Mei, Huan Li, Xinpeng Zhou, Min Cheng, Kele Cui

**Affiliations:** ^1^ Department of Thoracic Surgery, The First Affiliated Hospital of University of Science and Technology of China (USTC), Division of Life Sciences and Medicine, University of Science and Technology of China, Hefei, China; ^2^ Department of Thoracic Surgery, Anhui Provincial Hospital Affiliated With Anhui Medical University, Hefei, China; ^3^ Department of Thoracic Surgery, Anhui Provincial Hospital, Wannan Medical College, Hefei, China; ^4^ Department of Geriatrics, Gerontology Institute of Anhui Province, The First Affiliated Hospital of University of Science and Technology of China (USTC), Division of Life Sciences and Medicine, University of Science and Technology of China, Hefei, China; ^5^ Anhui Provincial Key Laboratory of Tumor Immunotherapy and Nutrition Therapy, Hefei, China; ^6^ Cancer Immunotherapy Center, The First Affiliated Hospital of University of Science and Technology of China (USTC), Division of Life Sciences and Medicine, University of Science and Technology of China, Hefei, China; ^7^ Department of Clinical Laboratory, The First Affiliated Hospital of University of Science and Technology of China (USTC), Division of Life Sciences and Medicine, University of Science and Technology of China, Hefei, China

**Keywords:** CD8^+^ Trm cells, characteristics, antitumor effects, immunotherapy, digestive tract tumors

## Abstract

Malignant digestive tract tumors are a great threat to human public health. In addition to surgery, immunotherapy brings hope for the treatment of these tumors. Tissue-resident memory CD8^+^ T (Trm) cells are a focus of tumor immunology research and treatment due to their powerful cytotoxic effects, ability to directly kill epithelial-derived tumor cells, and overall impact on maintaining mucosal homeostasis and antitumor function in the digestive tract. They are a group of noncirculating immune cells expressing adhesion and migration molecules such as CD69, CD103, and CD49a that primarily reside on the barrier epithelium of nonlymphoid organs and respond rapidly to both viral and bacterial infection and tumorigenesis. This review highlights new research exploring the role of CD8^+^ Trm cells in a variety of digestive tract malignant tumors, including esophageal cancer, gastric cancer, colorectal cancer, and hepatocellular carcinoma. A summary of CD8^+^ Trm cell phenotypes and characteristics, tissue distribution, and antitumor functions in different tumor environments is provided, illustrating how these cells may be used in immunotherapies against digestive tract tumors.

## Introduction

Malignant digestive tract tumors are a great threat to human public health. According to 2020 global cancer statistics, digestive tract tumors such as esophageal cancer (EC), gastric cancer (GC), colorectal cancer (CRC), and hepatocellular carcinoma (HCC) rank in the top 10 in cancer incidence and mortality and account for 23.4% of all new cases and 36.7% of deaths (1). The gastrointestinal mucosa is prone to inflammatory lesions and tumors resulting from long-term stimulation by physical and chemical factors and microorganisms (2). When tumors occur, although innate immune cells, as the vanguard, can induce rapid effector responses, powerful adaptive immunity involving various subsets of T cells, which is then triggered, is the main force to exert antitumor roles (3). As an important member of memory T cells, the tissue-resident memory T (Trm) subset is a group of noncirculating immune cells that reside in peripheral tissues and mediate tumor defense through cytokine secretion in humans and rodents (4–6). Trm cells include CD8^+^ Trm cells, CD4^+^ Trm cells, regulatory Trm cells, natural killer Trm cells, and γδ Trm cells, in which CD8^+^ Trm cells are extensively studied in antitumor research due to their powerful cytotoxic activity. CD8^+^ Trm cells mainly reside on the barrier epithelium of nonlymphoid organs and respond rapidly to both viral and bacterial infection and tumorigenesis. In human digestive tract mucosa, CD8^+^ Trm cells play a key role in anti-infection and antitumor immunity because they elicit a rapid immune response after antigen stimulation (7) .

Thus, CD8^+^ Trm cells play an important role in maintaining homeostasis and resisting tumorigenesis within the digestive tract mucosa. By recognizing homologous antigens, CD8^+^ Trm cells in the tumor microenvironment (TME) can rapidly secrete cytokines to activate innate immune cells and enhance the expression of chemokines and adhesion receptors, which in turn recruit circulating immune cells needed to exert essential antitumor functions. CD8^+^ Trm cell infiltration is associated with improved prognosis in common digestive tract tumors, such as EC, GC, CRC, and HCC (8–11).

Many treatments for malignant digestive tract tumors have shifted from traditional chemotherapy to a combination of chemotherapy and immunotherapy. In the TME of most digestive tract cancers, CD8^+^ Trm cells usually show an exhausted phenotype with the expression of inhibitory immune checkpoints such as programmed cell death protein-1 (PD-1) and T cell immunoglobulin and ITIM domain (TIGIT) (12–14). Although immune checkpoint inhibitors are widely used in the treatment of digestive tract tumors, there is still a high incidence of immune-related adverse events, and many patients do not respond well to immune checkpoint inhibitors due to the absence of prognostic markers, resulting in poor therapeutic outcomes (15–17). Therefore, adequate understanding of how variations in CD8^+^ Trm cells in the TME affect digestive tract tumor pathogenesis is of great practical significance for clinical treatment. However, until now, the roles of CD8^+^ Trm cells in digestive tract tumors have not been comprehensively described.

Herein, we review recent progress in understanding of the tissue distributions, biological characteristics and antitumor mechanisms of CD8^+^Trm cells in EC, GC, HCC and CRC to provide directions for combined precision targeted therapy strategies and prognosis prediction.

## Biological Characteristics of CD8^+^ Trm Cells

### The Origin and Maintenance of CD8^+^ Trm Cells

**Figure 1:**
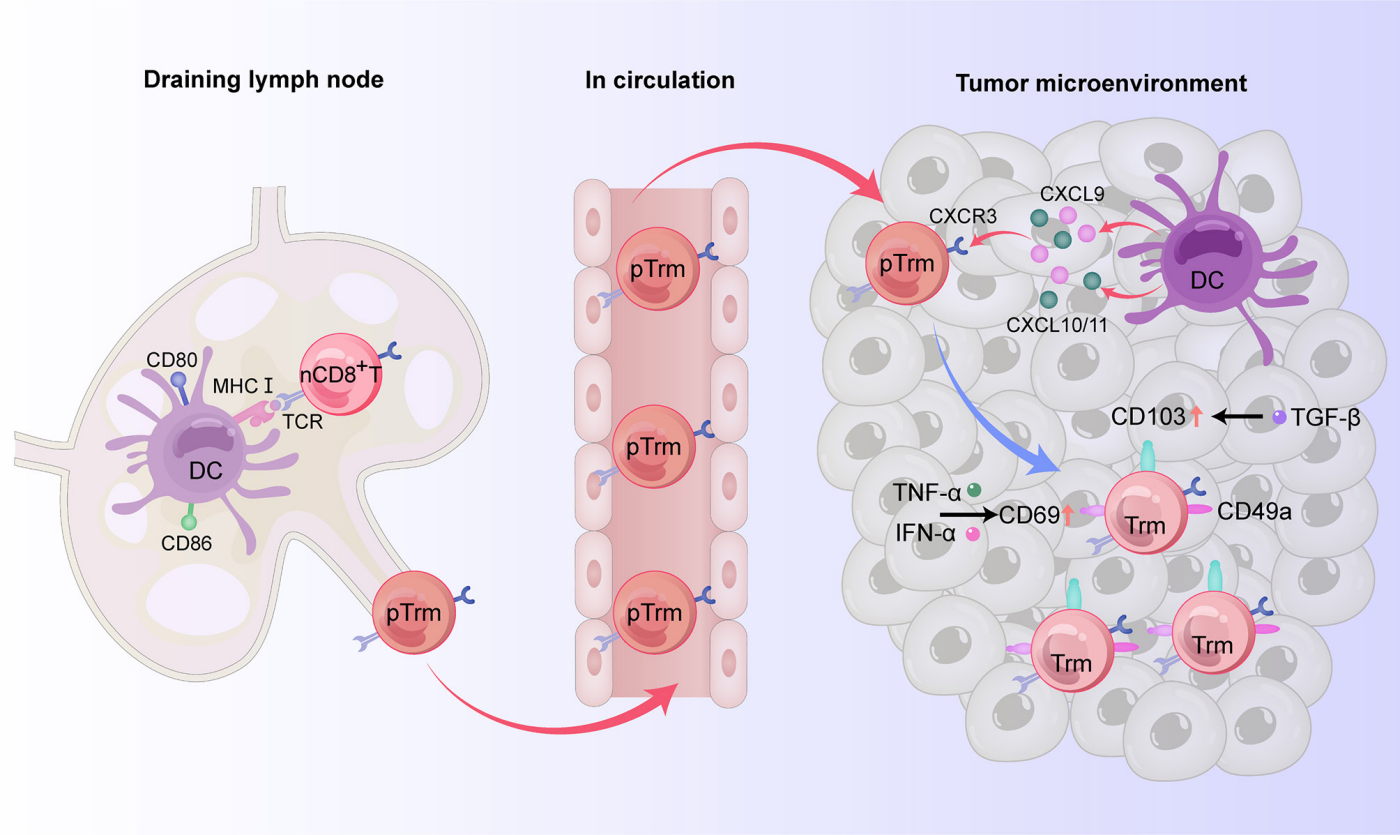
The origin and phenotypes of CD8^+^ Trm cells in human digestive tract tumors. In the draining lymph node, naive CD8^+^ T cells can differentiate into precursor Trm (pTrm) cells after cross-priming by Batf3-driven DCs and then enter the blood and circulate into the target tissue. By producing the CXCR3 ligands CXCL9 and CXCL10 (CXCL11 in humans), another subset of DCs remaining in the tumor site recruits pTrm cells into the tumor microenvironment. CD69 is upregulated on pTrm cells after exposure to TNF-α and IFN-α. After reaching the upper cortex, pTrm cells express CD103 and further differentiate in response to TGF-β. In addition to expressing CD69 and CD103, mature CD8^+^ Trm cells also express the adhesion molecule CD49a, thus possessing resident properties.

Trm cells are differentiated from naive T cells (18). The predominant phenotypes of CD8^+^Trm cells express CD69, CD103, and CD49a (19–21), but do not express lymphoid homing molecules CCR7 and CD62 L and cannot be recycled (22–24). For tumor immunity, cross-priming by type 1 classical dendritic cell (cDC1) subsets, whose development and/or function depends on basic leucine zipper ATF-like transcription factor 3 (Batf3) transcription, is necessary for optimal generation of Trm cells (25–27). Indeed, Batf3-lineage DCs migrate to the draining lymph node to mediate T cell cross-priming, while another subset remains in the tumor site to produce CXCR3 ligands CXCL9 and CXCL10 (CXCL11 in humans) used to recruit CD8^+^ effector T cells back to the target tissue (27). After cross-priming by Batf3-driven DCs, naive T cells and central memory T (Tcm) cells can differentiate into precursor Trm (pTrm) cells that enter the blood and circulate into targeted tissues. CD69 is upregulated on pTrm cells after exposure to IFN-α released by macrophages. After reaching the upper cortex, pTrm cells express CD103 and further differentiate in response to TGF-β. Kruppel-like factor 2 (KLF2) is a transcription factor encoding sphingosine-1 phosphate receptor 1 (S1PR1) and CD62 L, two molecules critical for naive T cell recirculation (28). Competition of CD69 and S1PR1 enables T lymphocytes to reside in peripheral tissue and differentiate into Trm cells. At the same time, T cells entering the epithelial tissue upregulate CD103 and downregulate the transcription factor KLF2 in response to TGF-β, promoting the residence of CD8^+^ T cells (29). TNF-α and type I interferon can upregulate the expression of CD69 on the surface of CD8^+^ Trm cells (24). In CD103^-^Trm cells, the memory lymphocyte cluster (MLC) can also provide signals to maintain CD103^-^Trm residence (23, 24) (**Figure 1**).

Although CD69 expression is upregulated in the early stage of Trm cell development, it cannot be used as a reliable marker of tissue residence because it is also expressed on other immune cells, and T cells expressing CD69 are still able to enter the circulation (30). CD103, also known as α E-integrin and human mucosal lymphocyte antigen, is an integrin expressed on intraepithelial T cells and some peripheral regulatory T cells. By binding to its ligand E-cadherin, CD103 can make antigen-specific T lymphocytes reside in epithelial tissue and is thus considered a reliable marker for Trm cells (23). CD49a, also known as very late antigen-1 (VLA-1), is a member of the integrin family. By binding to collagenase type IV, CD49a can prompt cells to be retained and survive in tissues (31). Furthermore, the maintenance of Trm cells in tissues is dependent on cytokines such as TNF-α, IL-15, TGF-β, and IL-33, while migration and retention are impacted by chemokines such as C-X-C motif chemokine receptor 6 (CXCR6), CCR10, and CXC chemokine ligand 17 (CXCL17) (30).

### The Role of CD8^+^ Trm Cells in the Antitumor Immune Response

Tumor-infiltrating CD8^+^ T cells are effector T cells that can directly recognize and kill target cells, serving as the immune system’s frontline force against tumors. CD8^+^ T lymphocytes are represented by cytotoxic T lymphocyte (Tc1) subsets, which have antitumor and anti-infection functions by producing high levels of perforin, granzyme B, IFN-γ, and TNF-α (32). Of the immune cells that infiltrate the TME, the infiltration of CD8^+^ T lymphocytes, especially Tc1 subsets, is usually associated with a more favorable prognosis (33). The antitumor function of CD8^+^ T cells depends on both differentiation and transport into the TME (34). In the TME of solid tumors, factors such as abnormal chemokine secretion and tumor angiogenesis can hinder the transport and function of CD8^+^ T lymphocytes (35). When this occurs, CD8^+^ Trm cells play an extremely important role in the antitumor process (36). Among the various subsets of Trm cells, CD8^+^ Trm cells are considered the first line of defense for peripheral tissues to inhibit early exposed antigens and have thus received considerable attention. The response of CD8^+^ Trm cells to re-exposed homologous antigens in the barrier tissue is faster than the response of circulating memory T cells (37, 38), primarily as a result of the critical locations in which they reside. These regions are the most common sites exposed to pathogens such as bacteria and viruses and where epithelial cancers originate. When activated, CD8^+^ Trm cells can quickly release perforin and granzyme B to directly kill target cells (6, 39) and amplify the activation of a small number of cells into an organ-wide response (40). While Trm cells may have phenotypic heterogeneity based on their location in the epithelia or stroma and the tumor subtype, these cells can promote recruitment of T lymphocytes into the epithelial TME and enhance the early signal transduction of CD8^+^ T lymphocytes within tumors (41). During tumorigenesis, CD69^+^CD8^+^/CD103^+^CD8^+^/CD49a^+^CD8^+^ T lymphocytes are highly activated, showing better effector function than traditional CD8^+^ T cells, and are able to control tumor growth (42).

**Figure 2:**
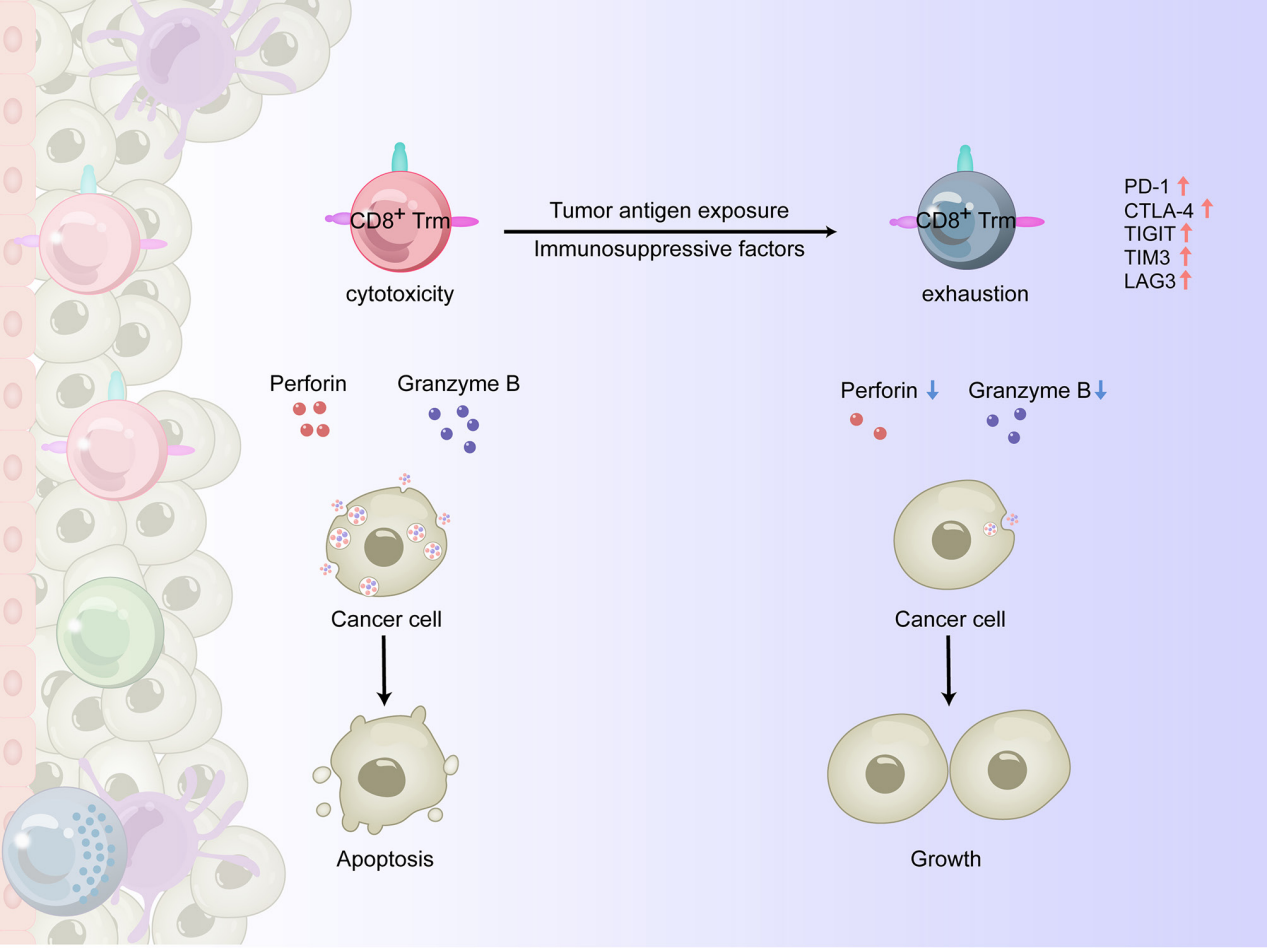
The antitumor effects of CD8^+^ Trm cells in the TME of human solid tumors. In the process of tumorigenesis, CD8^+^ Trm cells could be highly activated and show a higher effector function than traditional CD8^+^ T cells, releasing perforin and granzyme B and killing cancer cells. However, when persistently exposed to tumor antigens and immunosuppressive factors, the upregulation of inhibitory receptors such as PD-1, CTLA-4, TIGIT, TIM3 and LAG3 leads to impaired killing function and exhaustion of CD8^+^ Trm cells, making them unable to control tumor growth.

When persistently exposed to tumor antigens, upregulation of inhibitory receptors such as PD-1, cytotoxic T lymphocyte associated antigen-4 (CTLA-4), TIGIT, T cell immunoglobulin-and mucin-domain-containing molecule-3 (TIM3), and lymphocyte activation gene-3 (LAG3) can lead to impaired killing function and exhaustion of CD8^+^ T cells (43, 44). For example, as esophageal squamous cell carcinoma (ESCC) progresses, changes in the TME are accompanied by an increase in immunosuppressive cells such as regulatory T (Treg) cells, myeloid-derived suppressor cells (MDSCs), and immuno-suppressive DCs, as well as soluble inhibitory molecules such as indole-2,3 dioxygenase (IDO) (45) and fibroblast growth factor 2 (FGF2) (46), resulting in reduced infiltration and functional inhibition of CD8^+^ T cells (47). In recent years, it has been shown that tissue-resident T lymphocytes can overexpress PD-1 and other immune checkpoint molecules, such as TIGIT, LAG-3, and Tim-3, in some experimental animal and human tumor tissues (36, 48). There are two possibilities for this phenomenon: 1) tumor infiltrating CD8^+^ T lymphocytes express a variety of integrins, including CD49a, and remain in the TME in a quiescent/exhausted state, or 2) CD8^+^ T cells in the TME upregulate the expression of multiple integrins after exhaustion through an undetermined mechanism (**Figure 2**).

The following sections define the characteristics of CD8^+^Trm cells along with current research evaluating a role for CD8^+^Trm cells in antitumor therapy for four common digestive tract cancers, EC, GC, CRC, and HCC (**Table 1**).

**Table 1:** Characteristics of CD8^+^Trm cells in human digestive tract tumors.

Tumor types	Phenotypes	Inhibitory receptors	Cytotoxicity	Characteristics	Cytokines	References
EC	CD69 CD103	PD-1 TIGIT TIM-3	+	In addition to expressing inhibitory receptors, CD8^+^Trm cells in the EC have high proliferation ability and high cytotoxicity-related molecule expression.	IFN-γ IL-2 CD107a	(13, 49)
GC	CD69 CD49a CD103 RUNX3	PD-1 TIGIT CD39	+	CD8^+^Trm cells in the GC can induce SPEM by producing high levels of IFN-γ, produce high levels of cytolytic enzyme and IFN-γ in the presence of a large amount of various inhibitory receptors, and are related to the formation of TLS.	IFN-γ Granzyme B Perforin CD107a IL-2 TNF-α	(10, 21, 28, 50–53)
CRC	CD69 CD103	PD-1 CD39	+	CD8^+^Trm cells in the CRC have significant resident properties and tumor reactivity. With a unique methylome pattern and distinct epigenetic properties, they can enhance tissue immunity, improve barrier function, and prevent microbiota-associated diseases.	IFN-γ Granzyme B Perforin	(11, 54–59)
HCC	CD69 CD49a CD103 CD49b CD11c	PD-1 TIM-3 LAG-3 CTLA-4 CD244 CD39	+	As a unique population with low cytotoxicity, hepatic CD8^+^Trm cells provide long-term protection for human papillomavirus-like virus HPV-induced HCC.	Granzyme B Granzyme K Perforin Granulysin	(60–62)

## Characteristics of CD8^+^ Trm Cells and Their Potential Use in the Treatment of Digestive Tract Cancers

### CD8^+^ Trm Cells in EC

In 2020, EC ranked seventh in new cases and sixth among cancer-related deaths, with one in 18 deaths caused by EC (1). ESCC, which primarily occurs in Asian countries, accounts for about 90% of all pathological types of EC (63). Since it is directly exposed to foreign antigens in food, the esophageal mucosa has a special immune cell composition that plays an important role in maintaining esophageal homeostasis and mucosal anti-infective and antitumour processes. Strong expression of CD45RO, CD8, CD3, and CD107a in EC tissues indicates that there are cytotoxic memory CD8^+^ T cells in the stroma of these tumors (64). Although CD103^+^CD8^+^ T cells express PD-1 and TIM-3 in ESCC, they are relatively active cell subsets (12). Cells with the Trm phenotype have higher proliferation ability and express cytotoxicity-related molecules, indicating that there are highly activated antitumor subsets in CD8^+^ tumor infiltrating lymphocytes (TILs) in the TME.

The role of CD8^+^ Trm cells in EC is not well understood. Alterations in CD8^+^ Trm cell phenotypes and biological functions and the significance of these cells to EC prognosis and diagnosis remain obscure. Indeed, we have focused on the role of tissue-resident CD8^+^ T cells in EC for many years and found that CD49a, PD-1, and TIGIT molecules are highly expressed on CD8^+^ T cells in the TME of ESCC patients, indicating that there is also a population of tissue-resident CD8^+^ T cells with high expression of CD49a that shows the immune exhaustion phenotype in the ESCC TME. Multiple components of the ESCC TME can lead to immune exhaustion of CD103^+^CD8^+^ TILs, which can be repaired by αPD-1 blockers.

Clinical studies show that CD103^+^ CD8^+^ TILs are linked to the overall survival of ESCC patients (12). Thus, CD103 may be a suitable marker to evaluate the antitumor immune response of CD8^+^ T cells in ESCC, and infiltration of CD103^+^CD8^+^ TILs in the TME may be used as a biomarker to predict better prognosis in esophageal carcinoma (8, 12). It is worth noting that understanding the phenotype and function of CD8^+^ Trm cells in the occurrence and development of ESCC and exploring how best to reverse immune exhaustion and restore the antitumor function of CD8^+^Trm cells is an urgent issue that must be addressed by ESCC immunotherapeutic research. Establishing effective immune intervention strategies that target inhibitory molecules and reverse immune exhaustion will improve precision clinical immunotherapy for ESCC.

### CD8^+^ Trm Cells in GC

GC is one of the most common cancers in the world. In 2020, this disease ranked fifth in morbidity, with more than one million new cases, and fourth in mortality, with an estimated 769,000 deaths (1). *Helicobacter pylori* infection is a major risk factor for the development of chronic gastritis to GC (65, 66), but the exact role of inflammatory components in disease progression remains unclear. Two types of gastric metaplasia, intestinal metaplasia and spasmodic cleavage peptide expression metaplasia (SPEM), are precancerous lesions of human gastric adenocarcinoma (51). The accumulation of CD8^+^ Trm cells in the gastric mucosa involves the regulation of absent in melanoma 2 (Aim2), one of the key components of the inflammasome. Previous studies show that the lack of Aim2 can promote the accumulation of CD8^+^ Trm cells in chronic inflammatory gastric mucosa by preventing CD62 L and S1PR1 function (67). While the high levels of IFN-γ produced by gastric CD8^+^ Trm cells can induce SPEM (68), these cells have antitumor cytotoxicity when a tumor occurs (67).

CD103^+^CD8^+^ Trm cells in GC have similar phenotypes to those in other nonlymphoid tissues, including downregulation of lymph node homing-related molecules such as CD62 L, CCR7, and T cell factor 1 (TCF-1) and upregulation of tissue inhabitation promoting molecules such as CD69, CD49a, and Runt-related transcription factor 3 (RUNX3) (20, 31, 50, 52, 69, 70). Approximately 30% of TILs in GC are CD69^+^CD103^+^ Trm cells, which highly express the inhibitory receptors PD-1, TIGIT, and CD39 (53). However, CD103^+^CD8^+^ T cells can produce high levels of cytolytic enzymes and IFN-γ in the presence of a wide variety of inhibitory receptors (9). Moreover, PD-1 blockade effectively restored the function of CD103^+^CD8^+^ T cells but not CD103^-^CD8^+^ T cells. Thus, CD103^+^CD8^+^ Trm cells represent highly activated T cell subsets in GC and play an important role in inhibiting tumors (9).

Trm cell metabolism in GC tissues does not utilize glucose but relies on fatty acid oxidation to maintain cell survival, such that loss of fatty acids results in Trm cell death. GC cells outperform Trm cells at lipid uptake and may induce Trm cell death. Targeting PD-L1 can promote the survival of Trm cells by reducing the expression of fatty acid binding protein (Fabp)4 and Fabp5 in gastric tumor cells, increasing the expression of Fabp4/5 in Trm cells, and promoting lipid uptake by Trm cells (53). Thus, metabolic reprogramming may be an effective way to prolong the life span of GC Trm cells and enhance antitumor immunity, including CD8^+^ Trm cell survival. In addition, B cells in the tumor can form cell masses known as tertiary lymphoid structures (TLSs), which can induce immune cells to effectively recognize and attack cancer cells. In the gastric TME, TLSs are positively correlated with tumor-infiltrating CD8^+^Trm cells. Studies have indicated that Trm cells may be related to the formation of TLSs, and both may improve the outcomes of targeted therapy for PD-1 inhibitors in GC (71–73).

### CD8^+^Trm Cells in CRC

CRC ranks third in the world in incidence and second in mortality (1). As the organ with the largest interface with its environment, the gut is exposed to billions of antigens every day. The immune system needs to ensure tolerance to non-dangerous antigens and establish a strong immune response against potentially dangerous antigens (74). Immune cells are unevenly distributed in the gut. While CD8^+^ T cells (especially CD8^+^ Trm cells), monocytes, and CD19^+^ B cells are concentrated in the proximal colon, γδ T cells and NK cells are more abundant in the transverse colon, and CD4^+^ T cells and antibody-secreting cells are enriched in the distal colon and rectum (54). CD8^+^ T cells in the human intestinal tract are mainly Trm cells, which have CD103 and CD69 phenotypes and provide the first response to infection and tumors on the mucosal surface. TGF-β plays different roles in the formation and maintenance of Trm cells in the intestine. During secondary lymphoid organogenesis, TGF-β inhibits the migration of effector CD8^+^ T cells to the intestine, while during maintenance, TGF-β promotes the residence of CD8^+^ T cells (55). The regulatory function of Trm cells in the intestinal tract may be involved in intestinal homeostasis. It has been reported that promoting Trm and dendritic cell interactions can enhance tissue immunity, improve barrier function, and prevent microbiota-associated diseases (56). Due to the distinctiveness of the intestinal tract, CD8^+^ Trm cells have phenotypic and functional heterogeneity in response to infection and cancer, from pluripotent to differentiated, and show preferential protection at sites of imminent exposure to pathogens or persistent disease (75). In CRC, CD103 and CD69 are associated with immune recognition of Trm cells (57–59). CD103^+^CD39^+^CD8^+^ T cells have significant resident properties and tumor reactivity (10), with a unique methylome pattern in which the tumor reactivity markers CD39 and CD103 are specifically demethylated. This process provides these cells with distinct epigenetic properties (76).

CRC can be divided into microsatellite stable CRC (MSS) and high microsatellite unstable CRC (MSI-H). While tumor-infiltrating lymphocytes are abundant in MSI-H, which make up approximately 15% of CRCs, MSS CRC lacks tumor-infiltrating lymphocytes and is thus associated with a less favorable prognosis (77, 78). CD8^+^ Trm cell numbers were much higher in MSI-H than in MSS. Other studies show that deletion of the IL-15 gene, which is essential to maintaining intestinal Trm cells (79), is associated with poor prognosis, indicating that CD8^+^Trm cells play an important antitumor role in CRC. However, in MSI-H CRC, the expression of PD1 tended to increase in CD8^+^Trm cells, indicating that checkpoint inhibition therapy targeting Trm cells in MSI-H CRC may be of great significance (79).

### CD8^+^ Trm Cells in HCC

In 2020, primary HCC was the sixth most frequently diagnosed cancer, with more than 900,000 new cases, and the third leading cause of cancer mortality, with 830,000 deaths (1). This malignant tumor usually occurs in chronic inflammatory liver disease, such as fibrosis or cirrhosis, and is associated with certain risk factors, including hepatitis B virus (HBV), hepatitis C virus (HCV), alcohol abuse, and metabolic diseases (80, 81). Increased infiltration of cytotoxic T, NK, and NKT cells in the liver plays an active antitumor role in primary HCC. To avoid unnecessary activation of innate immune cells during continuous exposure to food and microbial-derived antigens, the liver needs to maintain a relatively immunotolerant environment. When immunogenic stimulation occurs, liver CD103^+^ dendritic cells express high levels of MHC-II, CD80 and CD86, which result in massive activation of CD8^+^ T cells (82). For example, HBV induces IFNγ^+^CD8^+^ T cells to upregulate CD69 and CD103 and induces liver CD8^+^ T cells to show the Trm phenotype *in situ* (83). The presence of T cells and cytotoxic cells in TILs correlates with a favorable prognosis of patients with HCC. More than 50% of these tumor-infiltrating lymphocytes express CD69 (84), and about 20-30% are positive for CD103, thus showing resident characteristics. However, unlike other tumors, only about 5% of human hepatic CD69^+^CD8^+^ T cells express CD103 (85). Recent studies have shown that hepatic CD8^+^ Trm cells adhere to the liver *via* LFA-1, and the residence of CD8^+^ T cells in the hepatic sinusoid depends on the LFA-1-I/CAM-1 interaction (86). However, chronic tumor antigen stimulation and immunosuppressive cells and their production in the TME can put Trm cells into a “dysfunctional state”. Targeting immune checkpoint molecules such as PD-1, TIM-3, LAG-3, and CTLA-4 can restore the dysfunction of Trm cells (87). However, hepatic CD8^+^ Trm cells are a unique population with low cytotoxicity (60), which may be related to the immunotolerant ecological properties of the liver. Thus, anti-PD-L1 or anti-PD-1 alone may not restore this dysfunction, and other agents, such as IL-2, may have a synergistic effect in improving the antitumor immunity of CD8^+^ Trm cells in HCC (87). In addition, the development and maintenance of tumor-specific CD8^+^ Trm cells induced by adenoviral vector immunization vaccine in the liver can provide long-term protection for human papillomavirus-like virus (HPV)-induced HCC and can enhance the formation of CD8^+^ Trm cells by targeting CTLA-4 (61). Thus, CD8^+^ Trm cells may also play an active role in tumor vaccine therapy for HCC.

## Application of CD8^+^ Trm Cells in Cancer Immunotherapy

The exhaustion phenotype of CD8^+^Trm cells in the TME does not prevent antitumor activity from being reactivated. *In vitro* studies of CD103^+^CD8^+^ T cells with high expression of PD-1 in lung cancer have shown that blocking the expression of PD-1 on these immune cells can restore their cytotoxicity against autologous tumor cells, suggesting that anti-PD-1 therapy may restore the killing function of CD8^+^ Trm cells toward autologous tumors (62). In the last few decades, anti-PD-1/PD-L1 therapies have shown remarkable efficacy in patients with malignant gastrointestinal neoplasms. For instance, the international randomized phase III KEYNOTE-181 and KEYNOTE-590 studies in EC patients showed that pembrolizumab provided a clinically meaningful overall survival (OS) benefit versus the control group (88, 89). Indeed, clinically meaningful improvements in overall response rate (ORR), progression-free survival and OS were observed in GC patients treated with pembrolizumab plus chemotherapy in the KEYNOTE-059 and KEYNOTE-062 trials (90, 91). However, although anti-PD-1 mAb is a promising approach for advanced GC patients, the response rate is still limited, with an ORR of only about 12.0% and a disease control ratio of about 34.7% (92). Although immunotherapy has produced durable responses in MSI-H CRC, with recent FDA approval of pembrolizumab in the first-line setting of metastatic CRC (93), MSS CRC has long been considered resistant to PD-1/PD-L1 blockade. However, combination therapy, such as co-inhibition of anti-PD-1 and STAT3 or regorafenib, a small molecule tyrosine kinase inhibitor, can elicit an effective antitumor response in a small subset of MSS CRC patients (49, 94). Disappointingly, the ORR of checkpoint inhibitors in HCC patients is only 15-20% (95). Recently, the Nivolumab (CheckMate-459) III phase trial failed to meet the primary endpoint, so an effective immunosuppressive therapy against HCC is still lacking (96).

There is no denying that the use of PD-1 inhibitors to reverse the exhaustion of immune cells such as CD8^+^ Trm cells, alone or with other checkpoint antibodies, has had controversial results. Due to tumor heterogeneity, a lack of reproducibility of results, and a complex scoring system, PD-L1 is not suitable as a predictive biomarker (97). While methods such as the combined positive score, which detects PD-L1 levels in tumors and lymphocytes, can be used clinically to evaluate patient response to PD-1/PD-L1-related inhibitors, their specificity for evaluating therapeutic impact is poor (98). Therefore, treatment options for patients with unresectable, locally advanced, or metastatic esophageal cancer are still limited, requiring the search for new predictive indicators and immunotherapy strategies (99).

Another way to increase the number of functional CD8^+^ Trm cells in tumors is by inducing their expansion using tumor vaccines. Studies demonstrate that vaccination can induce Trm cells in the tissue after natural infection and vaccination. For example, intravaginal immunization or systemic perfusion has been shown to boost vaginal mucosa by inducing Trm cells in the reproductive area (100). In addition, encoding respiratory syncytial virus mechanisms or recombinant cytomegalovirus vectors of Bacille Calmette-Guerin vaccine proteins for intranasal vaccination promotes immune cells to develop resident properties (101, 102). The vaccine-specific CD8^+^ T cell response can provide long-term protection against HPV-induced skin cancer and HCC but is dependent on the induction and accumulation of CD8^+^ Trm cells by blocking CTLA-4 early after immunization (61). Local radiotherapy by vaccination (103), which changes the expression of selectin, integrin, and chemokines, can also enhance the recruitment of resident CD8^+^ T lymphocytes in the tissue and tumor site.

## Perspective

CD8^+^ Trm cell infiltration plays a critical role in the antitumor immune response in the digestive tract. CD69, CD103, CD39, and CD49a are the key biomarkers of tumor-reactive CD8^+^ Trm cells and can be used as prognostic molecules for different digestive tract tumors (57, 59). However, CD8^+^ Trm cells that have infiltrated digestive tract tumors can also express immune checkpoint molecules such as PD-1, CTLA-4, TIGIT, TIM3, and LAG3, which can damage their killing function and cause immune exhaustion (104, 105). While targeted application of immune checkpoint inhibitors has achieved good results, the lack of immune markers and disparate responses to immune checkpoint inhibitors diminish the efficacy of treatment. Determining how best to increase the number and function of tumor-associated CD8^+^ Trm cells helps to maximize antitumor immunity. There is also great diversity among CD8^+^Trm cell phenotypes found in different digestive tract organs. For example, while PD-1^hi^ CD8^+^ Trm cells highly express cell adhesion and tissue positioning markers, including CD69 and integrins CD11c, CD49a, CD49b, and CD103 in HCC (87), CD103^+^CD8^+^ Trm cells express tissue residency-promoting molecules, such as CD69, CD49a, and RUNX3, in gastric cancer (9). CD103 is an important marker of CD8^+^ Trm in ESCC. ESCC patients with co-expression of PD-L1/TIM3 or PD-L1/TIGIT in CD8^+^ Trm cells have a lower survival rate than those expressing either marker alone (106). This may explain why only a small number of ECC patients benefit from treatment with PD-1 inhibitors. The absence of predictive indicators results in a high rate of immune-related adverse events in response to drugs targeting PD-1/PD-L1, with only a small number of patients showing positive outcomes. Nevertheless, a novel strategy to solve this problem is developing nanodrug delivery systems with a high drug loading capacity and targeting ability. It has been reported that biodegradable polymers such as poly (ursolic acid) are used as drug carriers for treating CRC and other cancers. The anticancer drug effectively loaded into poly(salicylic acid) nanoparticles shows ultrahigh blood vessel penetration, tumor penetration, and tumor accumulation due to the special prickly nanostructure (107, 108). Thus, the combination of a therapeutic polymer platform and immunotherapy to achieve precise targeted therapy may be a new attractive therapeutic strategy for treating digestive tract cancer.

In conclusion, alimentary tract neoplasms are a serious threat to human health. Immunotherapy for digestive tract tumors still has many problems, including blind treatment, side effects, and disparate individual responses. CD8^+^ Trm cells exist in various digestive tract tumors and are closely related to disease prognosis. However, current research on the utilization of CD8^+^ Trm cells in digestive tract tumors is still in the early stages. Thus, a comprehensive understanding of CD8^+^ Trm cell phenotypes and the characteristics of corresponding immune checkpoint molecules that are expressed in digestive tract tumors will be important to help guide accurate diagnosis and treatment of different tumor types. Specific drug therapy and tumor vaccine therapy that targets tumor-associated CD8^+^ Trm cells may become an important direction for antitumor research and tumor precision therapy.

## Author Contributions

XM performed the study design and drafted the manuscript. HL and XZ participated in the manuscript writing. MC obtained the funding, participated in the paper design, and contributed fruitful discussions. KC conceived the study and participated in the paper design and writing. All authors contributed to the article and approved the submitted version.

## Funding

The study was supported by Natural Science Foundation of Anhui Province, China (1708085MH182).

## Conflict of Interest

The authors declare that the research was conducted in the absence of any commercial or financial relationships that could be construed as a potential conflict of interest.

The editor YM declared a shared parent affiliation with the author(s) XM, MC, KC at the time of the review.

## Publisher’s Note

All claims expressed in this article are solely those of the authors and do not necessarily represent those of their affiliated organizations, or those of the publisher, the editors and the reviewers. Any product that may be evaluated in this article, or claim that may be made by its manufacturer, is not guaranteed or endorsed by the publisher.

## Glossary

**Table:**

AIM	activation inducer molecule
Aim2	absent in melanoma 2
Batf3	basic leucine zipper ATF-Like transcription factor 3
CCR7	chemokine receptor 7
cDC1	classical dendritic cell
CRC	colorectal cancer
CTLA-4	cytotoxic T lymphocyte associated antigen-4
CXCL17	CXC chemokine ligand 17
CXCR6	C-X-C motif chemokine receptor 6
EC	esophageal cancer
ESCC	esophageal squamous cell carcinoma
Fabp	fatty acid binding protein
FGF2	fibroblast growth factor 2
GC	gastric cancer
HBV	hepatitis B virus
HCC	hepatocellular carcinoma
HCV	hepatitis C virus
HPV	papillomavirus-like virus
IDO	indole-2,3 dioxygenase
KLF2	Kruppel-like factor 2
LAG3	lymphocyte activation gene-3
MDSCs	myeloid-derived suppressor cells
MLC	memory lymphocyte cluster
MSI-H	high microsatellite unstable CRC
MSS	microsatellite stable CRC
ORR	overall response rate
OS	overall survival
pTrm	precursor Trm
RUNX3	Runt-related transcription factor 3
S1PR1	sphingosine-1 phosphate receptor 1
SPEM	spasmodic cleavage peptide expression metaplasia
TCF-1	T cell factor 1
Tcm	central memory T
TIGIT	immunoglobulin and ITIM domain
TILs	tumor infiltrating lymphocytes
TIM3	T cell immunoglobulin-and mucin-domain-containing molecule-3
TME	tumor microenvironment
TLSs	tertiary lymphoid structures
Treg	regulatory T
Trm	tissue-resident memory T
VEGF	vascular endothelial growth factor
